# An Automated and High-Throughput Immunoaffinity Magnetic Bead-Based Sample Clean-Up Platform for the Determination of Aflatoxins in Grains and Oils Using UPLC-FLD

**DOI:** 10.3390/toxins11100583

**Published:** 2019-10-10

**Authors:** Zhihong Xuan, Jin Ye, Bing Zhang, Li Li, Yu Wu, Songxue Wang

**Affiliations:** Academy of National Food and Strategic Reserves Administration, No.11 Baiwanzhuang Str, Xicheng District, Beijing 100037, China; xzh@chinagrain.org (Z.X.); zb@chinagrain.org (B.Z.); ll@chinagrain.org (L.L.); wyu@chinagrain.org (Y.W.)

**Keywords:** Aflatoxins, immunoaffinity magnetic beads, automated and high-throughput sample clean-up, ultra-high performance liquid chromatography with fluorescence detection (UPLC-FLD).

## Abstract

Sample clean-up remains the most time-consuming and error-prone step in the whole analytical procedure for aflatoxins (AFTs) analysis. Herein, an automated and high-throughput sample clean-up platform was developed with a disposable, cost-effective immunoaffinity magnetic bead-based kit. Under optimized conditions, the automated method takes less than 30 min to simultaneously purify 20 samples without requiring any centrifugation or filtering steps. When coupled to ultra-high performance liquid chromatography with fluorescence detection, this new analysis method displays excellent accuracy and precision as well as outstanding efficiency. Furthermore, an interlaboratory study was performed in six laboratories to validate the novel protocol. Mean recovery, repeatability, reproducibility, and Horwitz ratio values were within 91.9%–107.4%, 2.5%–7.4%, 2.7%–10.6%, and 0.26%–0.90, respectively. Results demonstrate that the developed sample clean-up platform is a reliable alternative to most widely adopted clean-up procedures for AFTs in cereals and oils.

## 1. Introduction

Aflatoxins (AFTs) are secondary metabolites produced predominately by fungi such as *Aspergillus flavus*, *A. parasiticus,* and *A. nomius* under favorable temperature, moisture, and relative humidity [1]. AFTs are ubiquitous in nature and have many types, including aflatoxin B_1_(AFB_1_), aflatoxin B_2_ (AFB_2_), aflatoxin G_1_ (AFG_1_), and aflatoxin G_2_ (AFG_2_) [2]. AFTs are highly teratogenic and carcinogenic to humans and animals, AFB_1_ for example, is 10 times more toxic than potassium cyanide and is classified as class I carcinogen by the International Agency for Research on Cancer [3]. The global prevention and control of aflatoxins, therefore, is highly valued. Many countries have extremely low maximum limits (MLs) for AFTs in food. The MLs of AFB1 in China and European Union are 5–20 μg/kg and 2–12 μg/kg, respectively. Considering the huge risk AFTs have on public health, a rapid, accurate, and efficient analytical method is of great importance for the detection of AFTs in foodstuffs.

Sample clean-up is a vital step in AFTs analysis method, which can significantly affect the accuracy and precision of results, but is also the most time-consuming and error-prone step. Widely used clean-up methods in AFT analysis include solid-phase extraction (SPE) [4,5], dispersive liquid–liquid microextraction [6,7], QuEChERS [8,9,10], and diluting crude extract [11,12,13,14,15]. Immunoaffinity columns (IAC) exhibit many advantages against these methods, including high specificity, selectivity, and stability, which is why they are the most popular method for clean-up mycotoxin contaminants in foodstuffs [16].

According to the 2016–2018 FAPAS report on international Proficiency Testing, 70, 91, 84, and 97% of participants used IAC as sample clean-up method in rice, maize, peanut and animal feed, respectively. AOAC INTERNATIONAL, International standard organization (ISO) and other related official organizations recommend using IAC for sample clean-up prior to detection, as its advantages compare well with other current sample clean-up methods. However, IAC has tedious steps that are difficult to operate, requiring professional staff. IAC suffers from long pretreatment time and incurs high cost compare with other commonly clean-up techniques [17]. IAC practices are also incompatible with common automated procedures because gels can collapse as a result of high pressure. Therefore, alternatives to IAC that improve on operation times, labor, and costs, while maintaining good sample clean-up and stability, are highly desirable.

Recently, immunoaffinity magnetic beads (IMB) have emerged as a novel material for separation. IMB were synthesized by conjugating monoclonal antibodies (mAbs) with magnetic beads. The interactions between antigen and antibody are highly specific, so the IMB clean-up method possesses high specificity and selectivity. In addition, the large specific surface area and the dispersion properties of IMB greatly shorten equilibrium time and increase the interactions between the sorbent and target, which results in a higher extraction capacity and detection sensitivity. More importantly, IMB circumvent possible blockages because of their dispersed nature, which is a significant problem for IACs. IMB has been used in many scientific researches, such as cell screening [18], antibody purification [19,20], peptide and protein analysis [21,22,23,24], biochemical research [25,26], and food safety [27].

Therefore, IMB exhibit highly attractive characteristics, particularly for their high accuracy, easy-handling, and relatively low cost, which makes them an appealing alternative to IAC for AFT analysis. Some attempts have been made in this direction [26,27,28,29,30]. However, most were manual methods and requires centrifugation or filtering steps. Clearly, the combination of IMB with automated procedure according to the easy-handle by magnetic field, could generate new analytical approaches that outperform conventional IAC.

In this study, IMB are present as an excellent alternative clean-up material to IAC using a simple, home-made platform. Although some platforms for magnetic bead separation such as KingFisher^TM^ from Thermo Fisher Scientific are commercial available, their platforms are primarily focus on big molecules such as DNA, RNA, or Proteins which is not suitable for mycotoxins because of the incapable sampling volume and progress of procedure, furthermore, lack of the related clean-up kit. To overcome these limitations, our magnetic bead-based platform, for the first time, is shown to clean-up aflatoxin in an automated and high-throughput manner. Our new clean-up method avoids any centrifugation and filtering steps because of the fast sedimentation of extract residue and the dispersed magnetic beads. The novel clean-up system is able to automatically complete loading, washing, and elusion steps with IMB using magnetic stick with plastic coat. Overall the method supports easy automation and can be used to pretreat samples on a large scale with high accuracy and relatively low costs. The feasibility of this automated method was evaluated to detect trace aflatoxins in complex samples by ultra-high performance liquid chromatography with fluorescence detection (UPLC-FLD) via a single-laboratory study and collaborative study, demonstrating that this could successfully replace IAC for automated and high-throughput clean-up of AFT contaminants in grains and oils.

## 2. Results and Discussion

### 2.1. Synthesis and Characterization of IMB

**Figure 1:**
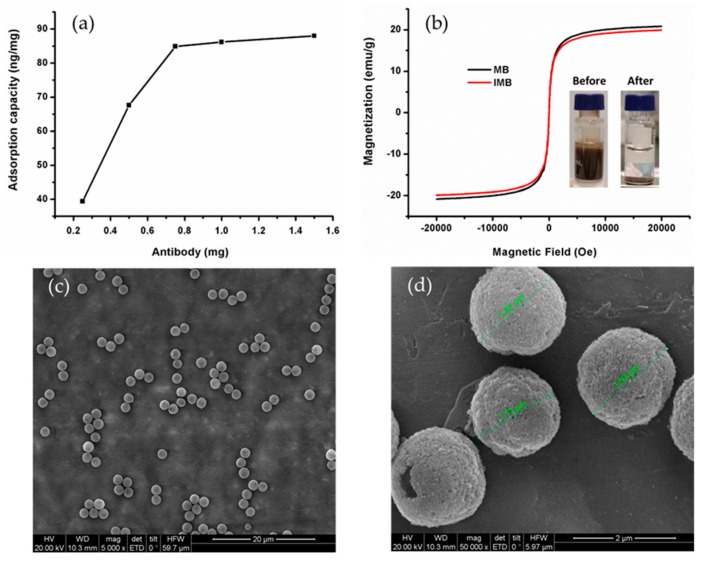
Synthesis and characterization of immunoaffinity magnetic beads (IMB). (**a**) Maximum binding capacity with different amounts of mAb. (**b**) Magnetization hysteresis loops spectrum and magnetic separation of IMB after adding the magnetic field. (**c**) and (**d**) SEM image of the obtained IMB.

To achieve a higher coupling efficiency of mAb, the maximum coupling amount of mAb on 10 mg of magnetic beads was optimized. The adsorption capacity corresponds to the maximum amount of AFT that can be retained, which is directly linked to the number of active mAb immobilized on the sorbent. To determine the adsorption capacity, 10 mg magnetic bead coupling with different amount mAb was reactive with 150 ng AFB_1_. Aa shown in Figure 1a, the adsorption capacity gradually increased as the antibody amount increased from 0.25 mg to 0.75 mg, which shows that the changes in the antibody amount affects the AFB_1_ adsorption capacity on the magnetic beads. When the antibody amount increased from 0.75 mg to 1.5 mg, the adsorption capacity tended to be stable, indicating that the antibody on the magnetic bead surface was already saturated. Therefore, 0.75 mg was deemed the most suitable antibody amount.

It is known that the IMB paramagnetism plays a key role in magnetic separation and can affect the accuracy of the proposed method. The magnetic properties of the magnetic beads and IMB were investigated by VSM (Figure 1b). Maximum saturation supermagnetizations of magnetic beads and IMB were measured at 20.82 and 19.90 emu/g, respectively. Although the addition of the nonmagnetic antibody led to decreased saturation supermagnetizations, the obtained IMB still displayed a high saturation supermagnetization of 19.90 emu/g. According to Ma’s study [31], a saturation supermagnetization of 16.3 emu/g is enough for magnetic separation from solution. The magnetic separation was achieved after adding the magnetic field (Figure 1b), IMB could be redispersed quickly with a slight shake once the magnetic field was removed, which indicates excellent magnetic responsivity and redispersibility. The morphology and particle size distribution of IMB were evaluated by SEM. Figure 1c,d show that the obtained IMB are spherical with a uniform particle size (2 μm) distribution. Furthermore, the lifetime of the IMB was also assessed. After a storage time of 12 months in a refrigerator at 4 °C, no significant differences in the recovery of AFT is observed (Figure S1), thereby revealing high stability of the IMB.

### 2.2. Automated Clean-up Platform

**Figure 2:**
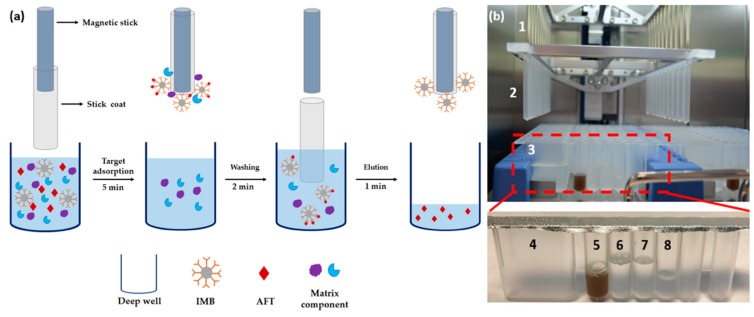
Automated clean-up platform (**a**) Schematic representation of the automated clean-up procedure using IMB. (**b**) Images of automated clean-up system, 1. Magnetic stick; 2. Stick coat; 3. disposable IMB kit; 4. Sample well; 5. IMB well; 6. Wash well; 7. Wash well; 8. Eluent well.

An IMB based automated clean-up system was constructed in-house by combing a magnetic stick with a plastic stick coat and a mechanical arm attached to the magnetic stick (Figure 2). The automated clean-up workflow is shown in Figure 2a, AFT are captured on the IMB by the antigen-antibody reaction in sample well. After the capturing step, the IMB are collected on the plastic coat through magnetic force offered by magnetic stick in the plastic coat and then transferred to the wash well through the control of a mechanical arm. After unwanted species are washed away, the IMB with the target are transferred and released to the eluent well by separating the magnetic stick from the plastic coat. The target analytes are then enriched in the elution solution and the IMB was discarded.

To further simplify the sample clean-up preceding, a disposable IMB based kit (Figure 2b) was designed as a near-complete package loaded with all the required IMB, dilution solution, wash solution, and elution solution. After loading extract of sample to the sample well, AFT was cleaned-up using the automated clean-up system. This automated clean-up platform rendered the clean-up processing simple, cost-effective, and self-contained; no additional reagents were needed for sample preparation. The cost of the IMB kit was about $5 per test (Table S1). These prices are expected go down with a scale-up production, which will give IMB competitive cost advantages over IAC ($15–$30).

### 2.3. Optimization of the Sample Clean-up Method

**Figure 3:**
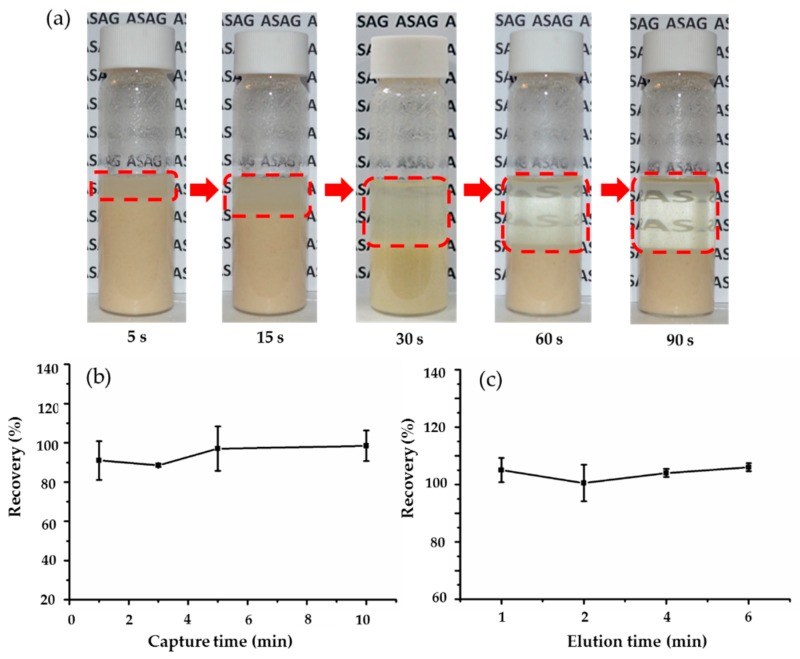
Optimization of sample clean-up methods. (**a**) Supernatant clarity as precipitate forms over time, (**b**) recovery in terms of capture time, (**c**) recovery in terms of elution time.

Commonly, centrifugation or filtering is the essential step due to the issue of packed bed clogging in IAC. In order to further simplify sample clean-up steps, a rapid sedimentation method was used rather than centrifugation or filtering after extraction. Figure 3a shows that it only takes 90 s to get a relatively clean supernatant for rapid sedimentation of husky rice flour extract residue. Although small particles possibly exist in the supernatant, magnetic beads are still fully functional and unobstructed. Other experiments also confirmed that the rapid sedimentation method was suitable for extraction from common cereals. Consequently, centrifugation or filtering was not required for the entire pretreatment process, which gives a simpler clean-up method to traditional IAC method.

In order to ensure all targets in the extract solution are captured by the IMB, capture time (1–10 min) was investigated. As shown in Figure 3b, recoveries of AFB_1_ were almost 100% when the capture time was only 5 min, which might be ascribed to the high affinity between the antibody on the IMB and targets in the extract solution. Hence, 5 min capture time was used for sample clean-up process in this study. In addition, the effect of elution time (1–6 min) was also evaluated. Figure 3c demonstrated that the recovery of AFB_1_ reached nearly 100% when the elution time was 1 min. Therefore, 1 min was deemed sufficient for target desorption. The automated clean-up processing was investigated according to previous optimization of the sample clean-up method. Under the optimal conditions, the automated method takes 28 min to purify 20 samples, whereas for manual IAC, almost 2 h is required.

### 2.4. Development of Clean-up of IMB Coupled to UPLC–FLD Detection

To establish a fast, convenient, and sensitive analytical method, UPLC–FLD was adopted. The sample obtained from automated clean-up through IMB was injected into UPLC and was separated on a C18 column and detected by FLD. The chromatographic separation conditions were investigated according to the response value, retention time, and resolution of AFT. The clean-up effect of the IMB to different grain and oil matrix was verified by the UPLC chromatogram (Figure S2). The comparison of the chromatogram between a standard solution and different matrix after IMB clean-up revealed that there was no interference from impurity peaks near the target chromatographic peaks.

### 2.5. Method Validation of Clean-Up of IMB Coupled to UPLC–FLD Detection

Analytical performances including linearity, sensitivity, accuracy and precision were validated by employing four commonly used cereal and oil matrices (maize, wheat, husky rice, and peanut oil).

#### 2.5.1. Linearity and Sensitivity

To establish the calibration curve, a series of working solutions (0.3125, 0.625, 1.25, 2.5, and 5.0 ng/mL) were prepared for the requirement of AFB_1_ and AFG_1_ analysis. For AFB_2_ and AFG_2_ analysis, a series of working solutions (0.078, 0.156, 0.3125, 0.625, and 1.25 ng/mL) were prepared. The calibration curves were then constructed by plotting the peak area (y) versus injected concentrations of standard solution (x), which resulted in the following four regression equations: y = 145682x + 1567.06 with a correlation coefficient (*r^2^*) of 0.9996 for AFB_1_; y = 127558x + 6013.87 with a correlation coefficient (*r^2^*) of 0.9998 for AFB_2_; y = 72872x + 375.55 with a correlation coefficient (*r^2^*) of 0.9999 for AFG_1_; y = 153116x + 3757.2 with a correlation coefficient (*r^2^*) of 0.9999 for AFG_2_; all demonstrated an excellent linearity. The standard curves are shown in Figure S3. The sensitivity of this method was estimated by the LODs and LOQs which were calculated from the spiked blank samples at different concentrations of AFT (AFB_1_, AFB_2_, AFG_1_, AFG_2_). The LODs, based on 3 times the signal-to-noise ratio, were in the range 0.03−0.1 μg/kg, and the LOQs, based on 10 times the signal-to-noise ratio, were in the range 0.1−0.3 μg/kg. The linearity range, LODs and LOQs are shown in Table S2.

#### 2.5.2. Accuracy and Precision

To evaluate the accuracy and precision of this method, recovery experiments were studied. Maize, wheat, husky rice and peanut oil samples were spiked with high, middle, and low concentrations of AFTs (10, 20, and 40 μg/kg for AFB_1_ and AFG_1_; 2.5, 5, and 10 μg/kg for AFB_2_ and AFG_2_). The results of the recovery and intra-day precision (expressed as RSD) are shown in Table 1. In all the cases, the recovery values were ranged from 85.1% to 109.3%, and RSD was lower than 7.4%, which fulfilled the performance criteria as set in European Commission Regulation (EC) No 401/2006 [32].

The accuracy of this method was also verified using certified reference material and reference materials. Four kinds of certified reference material or reference materials (maize, husky rice, peanut oil and rice) offered by ASAG and FAPAS were chosen here. Table 2 shows that all detection values for AFB_1_ were in the range of expanded uncertainty.

#### 2.5.3. Comparison between the IMB Automated Clean-up Method and the Conventional IAC Clean-up Method

In order to investigate the suitability of the automated clean-up method for the real-world samples, IMB was applied for the determination of AFB_1_ in 18 samples with different matrixes in China. The amounts of AFB_1_ that were determined by the IMB automated clean-up method were compared to those obtained by the traditional column-based method (Table S3). Figure S4 represents the two clean-up methods using different types of sample matrix were in agreement with a good fit (r^2^ = 0.997). Therefore, results indicate that the automated IMB pretreatment method is a promising alternative to the column-based IAC Method. In addition, the preparation of IMB is relatively safe while IAC require the use of highly toxic CNBr-activated sepharose-4B, which can produce highly toxic hydrocyanic acid, this adds further support for the use of IMB over IAC.

### 2.6. Interlaboratory Study

A Interlaboratory study was organized to evaluate the IMB automated clean-up coupled to UPLC–FLD method, six laboratories from China participated in the interlaboratory study, including Beijing Academy of Agricultural and Forestry Sciences, Beijing Cereal and Oil Food Inspection Institute, the Food inspection and Testing Institute of Henan Province, The Grain and Oil Quality Inspection Station of Guangxi Zhuang Autonomous Region, the Henan Cereal and Feed Product Quality Supervision and Testing Center and the monitoring station of the Sichuan Grain and Oil center. Spiked and naturally contaminated certified reference material or reference materials of husky rice and maize were used in these investigations. All participants returned their results by the deadline provided.

The results of the interlaboratory study were analyzed according to ISO 5725-2 [33]. Cochran and Grubbs tests were used to remove outliers. Table 3 and Table 4 show the statistical results obtained for the studied analytes. Mean recoveries of all tested aflatoxins were within the range of 91.9%–107.4%. RSDr values were in the range of 2.5%–7.4% and the RSD_R_ was within the range of 2.7%–10.6%, which is evidence that the developed IMB method has excellent repeatability and outstanding reproducibility. The average measured concentrations and SDs of AFB_1_ in certified reference material of maize and reference materials of husky rice were 28.5 ± 1.38 μg/kg and 26.8 ± 1.56 μg/kg. All were in good agreement with the (Certified) reference values. To evaluate the acceptability of precision of this method, HorRat values were calculated by comparing RSD_R_ with the respective predicted value at specific sample concentrations [34]. All HorRat values were <2.0, which is indicative of acceptable reproducibility precision. Overall, the recovery, repeatability, and reproducibility comply with the performance criteria of EU guidelines for AFTs [32]. The results from the interlaboratory study provided strong support for the utilization of IMB based methods for routine regulatory analysis of AFTs using automated, high-throughput, and reliable procedures reported here.

## 3. Conclusions

In conclusion, an appealing pretreatment method for clean-up AFTs from grains and oils was successfully developed through combining a disposable IMB-based kit with an in-house built automated system. Synthesis of IMB, capture time, and elution time were optimized. Method validation results showed that this method has a good linearity, precision, LODs, LOQs, recoveries and reproducibility using UPLC-FLD. In addition, results from an interlaboratory study indicate that this approach is a reliable clean-up method for quantifying AFT contamination. By consuming less time, cost and labor, as well as fewer errors from operation, the developed protocol overcomes the drawbacks of traditional column-based clean-up methods and thus offers a great prospect for AFTs analysis mainly in the applications of food safety and public health protection. The method can be easily extended to other mycotoxins by changing antibodies. Moreover, it is anticipated that this novel approach could be adapted for simultaneous detection of multiple mycotoxins and environmentally-friendlier practices.

## 4. Materials and Methods

### 4.1. Materials and Chemicals

HPLC grade methanol (MeOH) was purchased from Fisher Scientific (Atlanta, GA, USA). Ultra-pure water was obtained from a Milli-Q purification system (Bedford, MA, USA). Certificated standard solution of AFT containing 2 µg/mL of AFB_1_, 0.5 µg of AFB_2_, 2 µg of AFG_1_, and 0.5 µg of AFG_2_ in acetonitrile MeOH was obtained from Biopure (Tulln, Austria) and was stored at −20 °C. Working solutions were prepared by dilution with HPLC grade methanol, and then stored in vials at 4 °C and were renewed weekly. Tris(hydroxymethyl)aminomethane (Tris), sodium hydroxide, N-hydroxysuccinimide (NHS), N-(3-Dimethylaminopropyl)-N′-ethylcarbodiimide hydrochloride (EDC), and morpholinoethanesulfonic acid (MES) were purchased from Sigma-Aldrich (St. Louis, MO, USA). Carboxyl magnetic beads (10 mg/mL) and monoclonal antibody (mAb) of AFT were purchased from Kangyuan Techbio Biologicals (Beijing, China). Certified reference material and reference materials were obtained from Academy of National Food and Strategic Reserves Administration (ASAG) (Beijing, China; GBW (E) 100386, JTZK-001, JTZK-002, JTZK-007) and FAPAS (York, UK; Test no. 04335). Blank maize and husky rice samples were collected from a market in China.

### 4.2. Preparation of IMB

The IMB was prepared according to the previously reported [35] with a minor modification. A volume of 0.5 mL carboxyl magnetic beads were rinsed with 0.1 M MES (pH 4.5) three times. After the carboxyl magnetic beads were collected by a magnetic separator (Kangyuan Techbio Biologicals, Beijing, China), 0.5 mL of EDC (0.5 mg/mL) and 0.5 mL of sulfo-NHS (0.5 mg/mL) were added for the activation of carboxyl groups. After 30 min of shaking, the magnetic beads were separated by a magnetic separator and the supernatant was carefully discarded. The magnetic beads were then suspended in 0.5 mL mAb solution (0.75 mg/mL mAb in 0.1 M MES at pH 4.5) and incubated for 1 h with 500 rpm rotation at room temperature. After incubation, the supernatant was discarded, and beads were retained using a magnet against the tube. The magnetic beads were then suspended in 1 mL of blocking buffer (Tris-HCl, 0.01M, pH 8.0) to block the free active sites on the bead surface and incubated for 1 h with 500 rpm rotation at room temperature. After incubation, the supernatant was discarded, and beads were retained using a magnet against the tube. The precipitate was washed with PBST and PBS three times respectively. Finally, the obtained IMB can be stored in PBS at 4 °C for at least one year.

### 4.3. Characterization of IMB

The dried IMB were attached to the sample holder using a conducting glue and coated with 200 Å thick gold. The size and morphology of IMB were characterized by using scanning electron microscopy (SEM, Hitachi S-4800, Tokyo, Japan) which was operating at 20 kV. The magnetic property was determined by employing vibrating sample magnetometer (VSM, Quantum Design, MPMS-XL-7-SQUID) at room temperature.

### 4.4. Sample Extraction

Five grams of milled and thoroughly homogenized sample were weighed into a 50 mL centrifuge tube. Twenty milliliters of extraction solvent (methanol /water, 70:30, *v*/*v*) were added to the centrifuge tube and were mixed on a vortex shaker for 20 min. The centrifuge tube was then left to stand for 90 s to obtain the supernatant. The supernatants were collected for further analysis.

### 4.5. Automated IMB Clean-Up System

The automated clean-up system consisted of two mechanical arms and 20 permanent magnetic sticks. Two programmed stepping motors were used to move the magnetic sticks up and down or forward and backward. A kit holder and two stick coat holders were fabricated in the chamber of the platform. The system was fully controlled by a software supported by Kangyuan Techbio Biologicals (Beijing, China).

### 4.6. Automated IMB Clean-up Procedure and Optimization.

The IMB clean-up procedure was performed using an in-house built automated clean-up system. Supernatant (0.5 mL) obtained from the sample extraction and was diluted with 4.5 mL phosphate buffer (0.05 M, pH 7.4) containing 0.1% Tween 20 (*v*/*w*) (PBST) using an 8 mL deep well. The sequence of events involved in the sample clean-up was driven through the removal and separation of magnetic stick with plastic stick coat. The sequence of events is shown in Table S4.

Several key factors that may affect clean-up efficiency, such as capture time and elution time, were optimized. The capture time between IMB and AFB_1_ was investigated by incubating 1mg of MNPs with 1 ng / mL AFB_1_ standard solution that ranged from 1–10 min under 6 min of elution time. The elution time ranged from 1–6 min was investigated under optimized capture time.

### 4.7. Manual IAC Clean-up Procedure

The IAC clean-up procedures were according to the AOAC method [36] with some modifications. Fifteen milliliters of the supernatant obtained from sample extraction was diluted with 30 mL PBST solution. Thirty milliliters of diluted extract were collected and passed through the AFT IAC at 1–2 drops per second, followed by 10 mL of PBS solution. LC grade methanol (1.2 mL) was used to elute the AFT before diluting the eluate to volume with water and then perform LC analysis.

### 4.8. UPLC-FLD Analysis

A Waters ACQUITY UPLC H-class system (Waters, Manchester, UK) consisting of a vacuum degasser, an auto-sampler and binary pump, and a BEH reverse-phase C18 column (2.1 mm × 100 mm, 1.7 µm) were used for separation. the mobile phase consisting of methanol/ acetonitrile/water (17.5:17.5:65, *v/v/v*) with the flow rate of 0.2 mL/min; the excitation and emission wavelengths of fluorescence detector were 360 and 440 nm, respectively. Column and sample temperatures were maintained at 40 °C and 10 °C, respectively. The sample injection volume was 10 μL. Data were acquired and analyzed using Waters EMPOWER3 software.

### 4.9. Validation of Analytical Method

The automated pretreatment method prior to analysis with UPLC-FLD was evaluated for sample recovery, repeatability, linearity, limit of detection (LOD), and limit of quantitation (LOQ). Recovery studies were conducted by spiking with AFT standard solution at three different concentrations (high, intermediate, low) in the blank cereal and oil samples (wheat, maize, husked rice and peanut oil) in triplicates. Spiked samples were stored overnight at room temperature to evaporate the solvent from the spiked solution. In addition, trueness was assessed via testing certified reference material (GBW(E) 100386). Furthermore, repeatability was evaluated by experiments using spiked sample as well as the recovery study. Linearity was determined at five concentration levels. Calibration curves were created by plotting the relative peak areas of each compound versus the respective concentration. LODs and LOQs were calculated as the minimum concentration of mycotoxins that produced chromatographic peak area with signal-to-noise (S/N) ratio of 3 and 10, respectively. The LODs and LOQs values were determined using spiked blank samples of different concentrations.

### 4.10. Interlaboratory Study

Six laboratories participated in this interlaboratory trial. The participants were asked to strictly follow the protocol provided. The interlaboratory study used maize certified reference material and husky rice reference material and two different blank samples (maize and husky rice) for spiking. The samples were checked for homogeneity and were found to be sufficiently homogenous and stable according to the criteria of the 2006 International Union of Pure and Applied Chemistry’s International Harmonized Protocol [37].

### 4.11. Statistical Analysis

Statistical analysis of results, namely the elimination of outliers, evaluation of precision parameters (repeatability and reproducibility), means and Horwitz ratio (HorRat) values were based on the ISO 5725-2 standard [34].

## Figures and Tables

**Table 1:** Recovery and intra-day precision (RSD) for aflatoxins (AFTs) (*n* = 3). Abbreviations: Aflatoxin B_1_(AFB_1_), aflatoxin B_2_ (AFB_2_), aflatoxin G_1_ (AFG_1_), and aflatoxin G_2_ (AFG_2_).

	Maize			Wheat			Husked Rice			Peanut oil		
	Spiking Levels, μg/kg	Recovery, %	RSD, %	Spiking Levels, μg/kg	Recovery, %	RSD, %	Spiking Levels, μg/kg	Recovery, %	RSD, %	Spiking Levels, μg/kg	Recovery, %	RSD, %
AFB_1_	10	101.1	5.0	2.5	106.6	6.7	5	109.4	1.6	10	96.0	3.2
	20	97.8	1.6	5	100.6	0.1	10	103.3	4.1	20	100.0	6.2
	40	108.6	0.6	10	98.7	4.0	20	98.1	1.5	40	108.1	0.4
AFB_2_	2.5	94.2	4.1	0.625	105.6	7.1	1.25	109.3	2.4	2.5	92.7	2.3
	5	90.0	0.8	1.25	100.6	0.8	2.5	102.7	4.3	5	99.3	7.0
	10	92.0	0.8	2.5	99.8	5.4	5	97.2	1.3	10	108.9	2.4
AFG_1_	10	93.5	5.9	2.5	103.4	3.2	5	106.4	0.8	10	90.3	2.0
	20	93.5	1.4	5	100.6	1.2	10	102.7	1.1	20	96.6	5.5
	40	102.1	1.5	10	99.1	5.3	20	96.3	3.0	40	100.0	2.2
AFG_2_	2.5	95.0	6.1	0.625	98.0	7.4	1.25	103.9	1.4	2.5	90.8	2.1
	5	87.0	1.0	1.25	99.7	1.6	2.5	101.4	0.9	5	92.0	4.0
	10	91.9	1.5	2.5	98.4	4.8	5	96.7	2.7	10	85.1	3.2

**Table 2:** The detection results for the certified reference material and reference materials (*n* = 3).

Matrix	Test Number	Mycotoxin	Detection Value (μg/kg)	Certificate Value (μg/kg)	Range (μg/kg)
Maize	GBW(E)100386	AFB_1_	28.5	27	24–30
	FAPAS 04335	AFB_1_	5.6	4.6	2.57–6.62
Husky rice	JTZK-007	AFB_1_	26.84	26	22.1–29.9
Peanut oil	JTZK-002	AFB_1_	15.6	15.8	13.9–17.7
Rice	JTZK-001	AFB_1_	10.4	9.7	8.3–11.1

**Table 3:** Statistical analysis of interlaboratory study results for spiked maize and certified reference material (maize).

Sample	Spiking Levels (Low)				Spiking Levels (Medium)				Spiking Levels (High)				Certified Reference Material	
Toxin	AFB_1_	AFB_2_	AFG_1_	AFG_2_	AFB_1_	AFB_2_	AFG_1_	AFG_2_	AFB_1_	AFB_2_	AFG_1_	AFG_2_	AFB_1_	AFB_2_
Number of laboratories	6	6	6	6	6	6	6	6	6	6	6	6	6	6
Number of samples	18	18	18	18	18	18	18	18	18	18	18	18	18	18
Number of laboratories retained after eliminating outliers	6	6	6	6	6	6	6	6	6	6	6	6	6	6
Number of accepted results	18	18	18	18	18	18	18	18	18	18	18	18	18	18
Mean value/(μg/kg)	10.6	2.7	10.2	2.6	20.7	5.2	20.4	4.9	41.7	10.5	40.1	9.7	28.5	1.7
Repeatability standard deviation, Sr/(μg/kg)	0.56	0.09	0.60	0.16	0.82	0.17	0.99	0.14	1.86	0.52	2.11	0.54	1.38	0.11
Coefficient of variation of repeatability, C_v,r_ (%)	5.3	3.5	5.9	6.4	4.0	3.2	4.9	2.9	4.5	5.0	5.3	5.6	4.9	6.3
Repeatability limit r/(μg/kg)	1.59	0.26	1.71	0.46	2.33	0.47	2.80	0.40	5.28	1.48	5.96	1.54	3.92	0.30
Reproducibility standard deviation, S_R_/(μg/kg)	0.79	0.19	0.78	0.23	1.55	0.41	1.17	0.36	2.72	0.81	2.49	0.85	2.36	0.14
Coefficient of variation of Reproducibility, C_v,R_ (%)	7.5	7.1	7.6	8.9	7.5	7.8	5.8	7.2	6.5	7.7	6.2	8.8	8.3	8.3
Reproducibility limit R/(μg/kg)	2.23	0.54	2.20	0.64	4.39	1.16	3.32	1.01	7.70	2.30	7.04	2.40	6.68	0.39
HorRat value	0.67	0.52	0.68	0.64	0.74	0.63	0.57	0.58	0.72	0.69	0.68	0.78	0.86	0.56
Recovery (%)	105.8	107.4	102.2	102.2	103.6	104.6	102.0	98.7	104.1	105.2	100.2	96.6	-	-

**Table 4:** Statistical analysis of interlaboratory study results for spiked husky rice and reference materials (husky rice).

Sample	Spiking Levels (Low)				Spiking Levels (Medium)				Spiking Levels (High)				Reference Material	
Toxin	AFB_1_	AFB_2_	AFG_1_	AFG_2_	AFB_1_	AFB_2_	AFG_1_	AFG_2_	AFB_1_	AFB_2_	AFG_1_	AFG_2_	AFB_1_	AFB_2_
Number of laboratories	6	6	6	6	6	6	6	6	6	6	6	6	6	6
Number of samples	18	18	18	18	18	18	18	18	18	18	18	18	18	18
Number of laboratories retained after eliminating outliers	6	6	6	6	6	6	6	6	6	6	6	6	6	6
Number of accepted results	18	18	18	18	18	18	18	18	18	18	18	18	18	18
Mean value/(μg/kg)	5.0	1.3	4.6	1.2	10.4	2.6	9.9	2.5	20.1	5.0	18.9	4.8	26.8	1.4
Repeatability standard deviation, Sr/(μg/kg)	0.26	0.09	0.31	0.09	0.51	0.16	0.60	0.17	0.50	0.15	0.66	0.17	1.56	0.1
Coefficient of variation of repeatability, C_v,r_ (%)	5.2	6.8	6.7	7.0	5.0	6.0	6.1	6.8	2.5	2.9	3.5	3.5	5.8	7.4
Repeatability limit r/(μg/kg)	0.75	0.25	0.88	0.24	1.45	0.45	1.70	0.48	1.41	0.41	1.88	0.47	4.43	0.3
Reproducibility standard deviation, S_R_/(μg/kg)	0.39	0.12	0.48	0.11	0.58	0.21	0.65	0.17	0.54	0.26	1.10	0.18	2.35	0.14
Coefficient of variation of Reproducibility, C_v,R_ (%)	7.7	9.6	10.6	8.8	5.6	7.8	6.5	6.8	2.7	5.2	5.8	3.7	8.7	9.6
Reproducibility limit R/(μg/kg)	1.10	0.35	1.37	0.30	1.65	0.59	1.84	0.48	1.52	0.74	3.10	0.50	6.64	0.38
HorRat value	0.62	0.63	0.83	0.57	0.50	0.57	0.58	0.49	0.26	0.41	0.57	0.29	0.90	0.63
Recovery (%)	100.5	102.5	91.9	97.5	103.7	105.7	99.4	100.0	100.5	100.7	94.4	96.3	-	-

## Supplementary Materials

The following are available online at https://www.mdpi.com/2072-6651/11/10/583/s1, Figure S1: Recovery of AFB1 in terms of storage time, Figure S2: The UPLC chromatographic peaks comparison between the standard solution (AFT, 20 µg/kg) and the four kinds of grains and oil (maize, wheat, husky rice, peanut oil, 20 µg/kg, respectively) matrix pretreatment through the automated clean-up method, Figure S3: Calibration curves and correlation coefficients for AFT, Figure S4: Comparison of IMB and IAC methods for the determination of AFB1 in real-world samples, Table S1: Cost breakdown for IMB, Table S2: The linearity range, LOD, and LOQ for AFTs, Table S3: The concentration level of AFB1 (µg/kg) determined in rice, peanut oil and Maize using IMB and IAC method, Table S4: The sequence of events in the automated clean-up program.
