# Supplementary material for: An Automated and High-Throughput Immunoaffinity Magnetic Bead-Based Sample Clean-Up Platform for the Determination of Aflatoxins in Grains and Oils Using UPLC-FLD
Source: Toxins (Basel). 2019 Oct 10;11(10):583. doi: 10.3390/toxins11100583 (PMC6832433; doi:10.3390/toxins11100583)
Supplement: Supplementary file 1 [file toxins-11-00583-s001.pdf]

Supplementary Materials:An automated and high-throughput immunoaffinity magnetic bead-based sample clean-up platform for the determination of aflatoxins in grains and oils using UPLC-FLD

Zhihong Xuan, Jin Ye*, Bing Zhang, Li Li, Yu Wu, Songxue Wang*

Table S1. Cost breakdown for IMB.

Componet	Price ($)	Total Amount	Amount /test	# of tests	Cost/ test ($)
Carboxyl magnetic beads	1691.2	1000 mg	1 mg	1000	1.7
monoclonal antibody (mAb) of AFT	2941.2	100 mg	75 μg	1333	2.2
Deepwell strip	326.5	1000 pairs	1 pair	1000	0.3
Others (Solution and reagent)	-	-	-	-	0.8
Total	-	-	-	-	5.0

Table S2. The linearity range, LOD, and LOQ for AFTs.

Mycotoxin	Linearity (μg/kg)	LOD (μg/kg)	LOQ (μg/kg)
AFB_1	0.5–20	0.1	0.3
AFB_2	0.125–5	0.03	0.1
AFG_1	0.5–20	0.1	0.3
AFG_2	0.125–5	0.03	0.1

Table S3. The concentration level AFB_1 (μg/of kg) determined in rice, peanut oil and Maize using IMB and IAC method.

Sample	Matrix	IMB	IAC	Sample	Matrix	IMB	IAC
#1	Rice	10.5	10.2	#10	Peanut oil	16.5	16.4
#2	Rice	11.0	10.5	#11	Peanut oil	15.0	15.2
#3	Rice	10.0	9.8	#12	Peanut oil	15.3	15.8
#4	Rice	10.4	10.0	#13	Maize	31.0	31.7
#5	Rice	9.0	8.9	#14	Maize	33.0	32.4
#6	Rice	8.9	9.1	#15	Maize	31.0	31.5
#7	Peanut oil	15.5	15.1	#16	Maize	30.8	30.0
#8	Peanut oil	16.0	15.5	#17	Maize	30.6	31.0
#9	Peanut oil	15.7	15.9	#18	Maize	29.0	28.9

Table S4. The sequence of events in the automated clean-up program.

Step	Well	Mixture time/min	Collection time/min
IMB transfer	2	1	0.5
Incubation	1	5	0.5
Wash 1	3	1	0.5
Wash 2	4	1	0.5
Elution	5	1	0.5
IMB collection	2	1	0.5

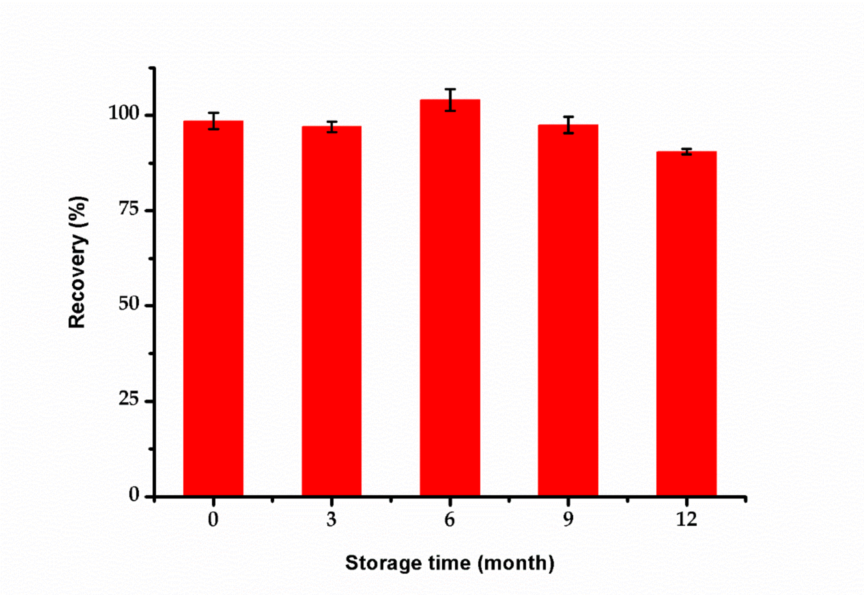

Figure S1. Recovery of AFB_1 in terms of storage time.

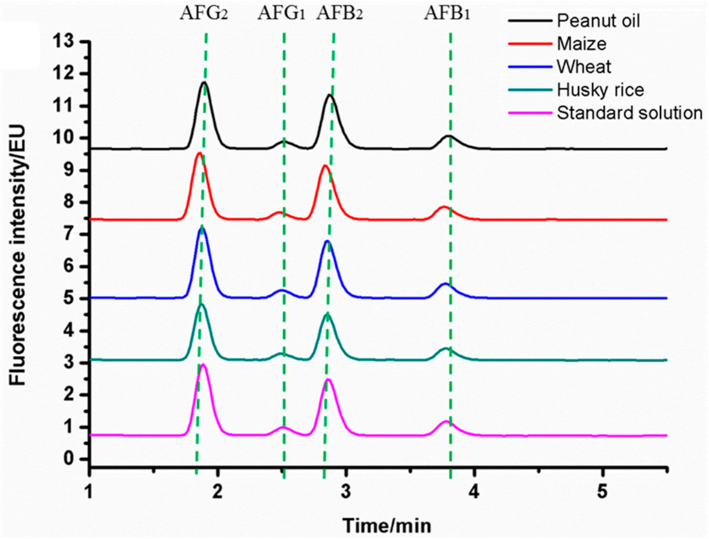

Figure S2. The UPLC chromatographic peaks comparison between the standard solution (AFT, 20 μg/kg) and the four kinds of grains and oil (maize, wheat, husky rice, peanut oil, 20 μg/kg, respectively) matrix pretreatment through the automated clean-up method.

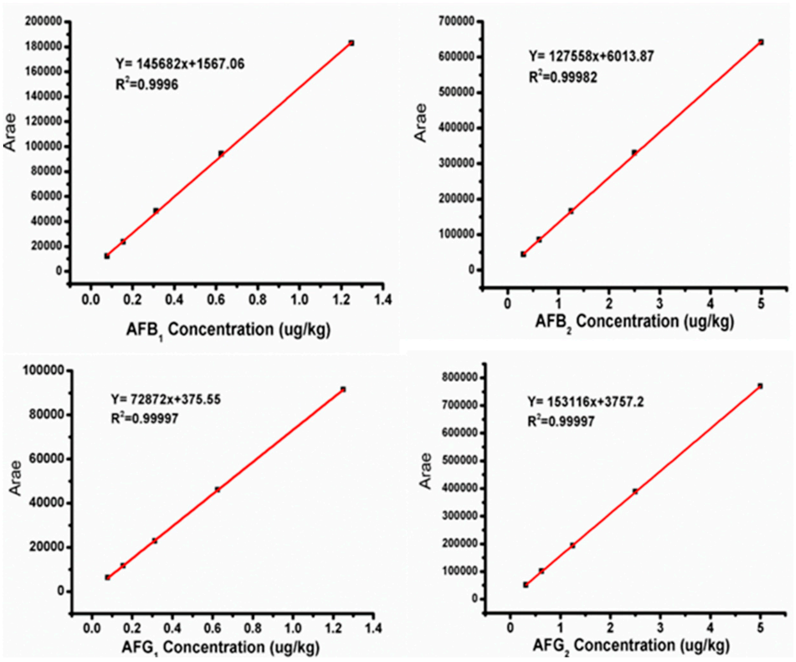

Figure S3. Calibration curves and correlation coefficients for AFT.

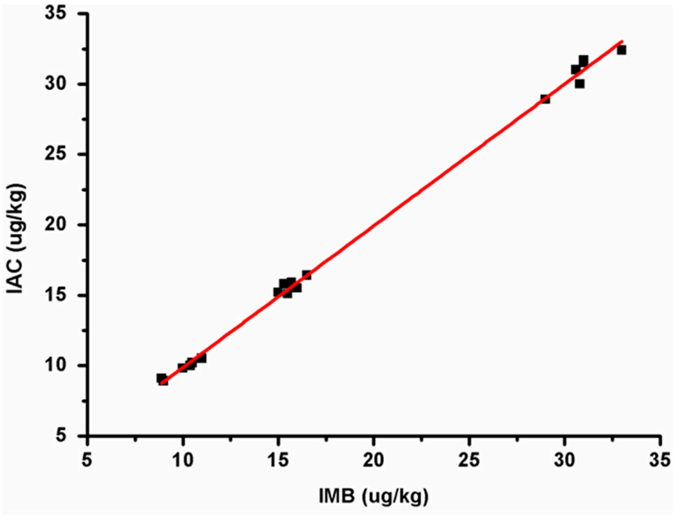

Figure S4. Comparison of IMB and IAC methods for the determination of AFB_1 in real-world samples.
